# The effect of anatomical factors on mortality rates after endovascular aneurysm repair

**DOI:** 10.5830/CVJA-2015-057

**Published:** 2016

**Authors:** Ay Derih, Erdolu Burak, Yumun Gunduz, Aydin Yumun, Demir Ahmet, Ozkan Hakan, Tiryakioglu Osman, Erkoc Kamuran

**Affiliations:** Department of Cardiovascular Surgery, Bursa Yüksek İhtisas Education and Research Hospital, Bursa, Turkey; Department of Cardiovascular Surgery, Bursa Yüksek İhtisas Education and Research Hospital, Bursa, Turkey; Department of Cardiovascular Surgery, Bursa Yüksek İhtisas Education and Research Hospital, Bursa, Turkey; Department of Cardiovascular Surgery, Bursa Yüksek İhtisas Education and Research Hospital, Bursa, Turkey; Department of Cardiovascular Surgery, Yalova State Hospital, Yalova, Turkey; Department of Cardiovascular Surgery, Bahcesehir University Medical Faculty and Medical Park Bursa Hospital, Bursa, Turkey; Department of Cardiovascular Surgery, Bahcesehir University Medical Faculty and Medical Park Bursa Hospital, Bursa, Turkey; Department of Cardiovascular Surgery, Medical Park Bursa Hospital, Bursa, Turkey

**Keywords:** abdominal aortic aneurysm, EVAR, endoleak

## Abstract

**Objective:**

The objective of this study was to investigate the effect of anatomical characteristics on mortality rates after endovascular aneurysm repair (EVAR).

**Methods:**

We investigated 56 EVAR procedures for infrarenal aortic aneurysms performed between January 2010 and December 2013, and the data were supplemented with a prospective review. The patients were divided into two groups according to the diameter of the aneurysm. Group I (*n* = 30): patients with aneurysm diameters less than 6 cm, group II (*n* = 26): patients with aneurysm diameters larger than 6 cm. The pre-operative anatomical data of the aneurysms were noted and the groups were compared with regard to postoperative results.

**Results:**

There were no correlations between diameter of aneurysm (*p* > 0.05), aneurysm neck angle (*p* > 0.05) and mortality rate. The long-term mortality rate was found to be high in patients in whom an endoleak occurred.

**Conclusion:**

We found that aneurysm diameter did not have an effect on postoperative mortality rates. An increased EuroSCORE value and the development of endoleaks had an effect on long-term mortality rates.

## Objective

An aortic aneurysm is defined as a greater than 50% increase in the aortic diameter compared to the normal proximal aorta.^1^ Large aortic aneurysms tend to enlarge and rupture eventually. The annual risk of rupture of aneurysms with diameters larger than 6 cm is more than 25%.^2^

Generally, elective surgery or endovascular repair is recommended in aneurysms with diameters larger than 5.5 cm, whereas in those with smaller diameters, follow up with ultrasonography or computerised tomography (CT) is recommended.^3^ Endovascular repair compares favourably to open aneurysm repair, with a significant reduction in morbidity, reduced blood loss, shorter hospital stay, and earlier return to normal function.^4^

The size of the abdominal aortic aneurysm is the major determinant of risk of aneurysm rupture and long-term survival.^5^ The threshold value for differentiation of small and large aneurysms is generally 5.5 cm. Aneurysms whose diameter is smaller than 5.5 cm are evaluated as small aneurysms and when they are greater than 5.5 cm, they are regarded as large aneurysms.

The treatment of AAA smaller than 5.5 cm with EVAR requires less recurring intervention compared with large aneurysms.^6^ Although early and long-term results in small aneurysms in which EVAR was performed were better when compared to large aneurysms, the relationship between size of the aneurysm and results after EVAR is unclear.^7^

The effect of EVAR on aneurysm morphology is changing. During or post-EVAR, it is possible that an angulated aneurysm neck can be straightened under the influence of the guidewire, the delivery system, and the stent-graft, and the aneurysm sac shrinks as a result.^8^

The aim of the current study was to determine the effect of pre-operative diameter and anatomical characteristics of the aneurysms on the outcome after EVAR. Therefore, patients with aneurysms larger than 5.5 cm were selected for the study and divided into two groups according to increased rupture risk (≤ 6 cm vs > 6 cm).

## Methods

A total of 56 patients underwent EVAR for a fusiform infrarenal AAA between January 2010 and December 2013 at Bursa Yuksek İhtisas Education and Research Hospital and Medical Park Bursa Hospital. Upon pre-operative evaluation, coronary artery disease was detected in 18 cases (32%), previous coronary artery bypass surgery was reported in eight (14%), and peripheral artery disease was found in eight cases (14%) (Table 1).

**Table 1:** Pre-operative characteristics of the patients

**	*Aneurysm ≤ 6 cm (group I)*	*Aneurysm > 6 cm (group II)*	*p-value*
Number (%)	30 (53.5)	(46.5)	> 0.05
Age (years)	68.5	72.1	> 0.05
Gender (M/F)	24/6	23/3	> 0.05
COPD, n (%)	16 (53)	18 (69)	0.02*
Smoking, n (%)	12 (40)	15 (58)	> 0.05
DM, n (%)	8 (26.6)	11 (20)	> 0.05
PAD, n (%)	2 (6.6)	6 (23)	0.001*
CABG, n (%)	3 (10)	5 (19)	0.02*
CAD, n (%)	10 (33)	8 (30.7)	> 0.05

COPD: chronic obstructive pulmonary disease, DM: diabetes mellitus, PAD: peripheral artery disease, CABG: coronary artery bypass graft, CAD: coronary artery disease. *Statistically significant.

The patients, all of whom were symptomatic, were divided into two groups according to the diameter of the aneurysm, measured with abdominal CT (at its largest site): group I included patients with an aneurysm diameter of 6 cm and below, and group II included patients with an aneurysm diameter larger than 6 cm. The patient data were prospectively collected. The primary end-point criteria of the study were determined as EVAR-related morbidity, and death of the patient. Prior to the operation, we measured maximum aneurysm diameters and neck, as well as common femoral and iliac maximal diameters in all CT axial slices.

In all patients, EVAR was performed via the main femoral artery route. In 11 (20%) cases, local anaesthesia, and in 34 (60%) cases, spinal and/or epidural anaesthesia were administered. General anaesthesia was administered in 11 cases (20%). In five of these cases the surgery began under local anaesthesia and then switched to general anaesthesia.

The mean age of the patients in whom aneurysm repair was performed with endovascular graft was 70.4 years (52–82); nine patients were female and 47 were male. The following types of grafts were implanted: in 31 cases Medtronic Endurant, in 19 Vascutek Anaconda, in three Trivascular Ovation, in two Gore Excluder, and in one case Lombard Aorfix (Table 2).

**Table 2:** Anatomical features of aneurysms

	*Aneurysm ≤ 6 cm (group I)*	*Aneurysm > 6 cm (group II)*	*p-value*
Graft Endurant	14	17	> 0.05
Anaconda	12	7	0.01*
Ovation	3	–	0.001*
Excluder	1	1	> 0.05
Aorfix	–	1	> 0.05
Mean diameter (cm)	5.8	7.79	0.001*
Neck angle (°)	63	67	0.04*
Neck length (cm)	1.7	1.65	> 0.05
Right iliac angle (°)	87	95	> 0.05
Left iliac angle (°)	95	90	> 0.05
Endoleak, n (%)	4 (13.3)	7 (27)	0.02*
Femorofemoral cross-over bypass, n (%)	1 (3.3)	3 (11.5)	0.02*
Ruptured aneurysm, n (%)	0 (0)	4 (9.09)	0.001*

*Statistically significant.

Thirty (53.5%) patients were in group I and 26 (46.5%) were in group II. The mean aneurysm diameter of patients in general was calculated as 6.6 cm (4.5–10.5 cm). The mean aneurysm diameter in group I was 5.8 cm (4.5–6.0 cm), and in group II it was 7.8 cm (6.1–10.5 cm) (Table 2). The number of the patients on whom urgent intervention was performed due to perforated aneurysm was four (9.09%) and the success rate of treatment was 100%.

## Statistical analysis

All data were expressed as mean and standard deviation using the SPSS 15.0 statistical program. The correlations between aneurysm diameter and mortality rate, and between neck length and endoleak were compared using logistic regression, and the other correlations were compared using the chi-squared test; *p* < 0.05 was accepted as significant.

## Results

In four cases (7%), aorta–uni-iliac EVAR was performed and an additional femorofemoral extra anatomical bypass was carried out. In all other cases the EVAR graft was placed aorta–bi-iliac. In one case, renal stent implantation was performed in the same session. In one patient, surgery was performed after the procedure to control bleeding due to iliac perforation.

In eleven cases (20%) an endoleak was detected during the procedure. Type I endoleak was detected in eight cases, seven of which were resolved after balloon application, and one was fixed with an aortic extension graft. In two cases, type II endoleak was detected; in one of these cases the causative vessel was occluded and in the other the leak was accepted as insignificant and followed up. Type IV endoleak was detected in one case and it disappeared during follow up.

In two cases (4.54%) renal failure was observed in the early period after EVAR. One of the patients returned to normal after a six-month period of haemodialysis, whereas the other continues life dependent on haemodialysis.

The mean duration of follow up of the patients included in the study was 48 months for group I and 55 months for group II. During the long-term follow up, two graft thromboses, one graft migration, three endoleaks and one case of mesenteric ischaemia were detected. Additional intervention was required in three patients. In-hospital deaths were observed in four patients and death occurred in a total of six patients (10.7%) (Table 3).

**Table 3:** Results during follow up

	*Group I*	*Group II*	*p-value*
Mean duration of follow up (months)	48	55	> 0.05
Graft thrombosis (single leg)	2	–	> 0.05
Graft migration	–	1	> 0.05
Endoleak	1	2	> 0.05
Mesenteric ischaemia (n)	–	1	> 0.05
Additional intervention	2	1	> 0.05
Mortality, n (%)*	3 (10)	3 (11.5)	> 0.05

*Total mortality rate in hospital and during the follow up.

The mean EuroSCORE of all the patients was calculated as 4 (1–9), and the mean EuroSCORE of the patients who died was 7 (4–9). In the statistical analysis, which was performed using the logistic regression method, no significant correlation was found between group I and group II in terms of aneurysm diameter and mortality rate. The increase in aneurysm diameter had no effect on mortality rate (*p* > 0.05) (Table 4). The mortality rate of the patients who had ruptured aneurysms was not different from the patients with non-ruptured aneurysms (*p* = 0.4).

**Table 4:** Effects of anatomical data on mortality rate

	*Deaths (n = 6)*	*Survivals (n = 50)*	*p-value*
Mean age (years)	72.1	69.5	> 0.05
Aneurysm diameter (cm)	6.9	6.7	> 0.05
Neck angle (°)	66	65	> 0.05
Neck length (cm)	1.5	1.69	> 0.05
Right iliac angle (°)	90	92	> 0.05
Left iliac angle (°)	92	90	> 0.05
Endoleak, n (%)	6 (100)	5 (10)	0.001*
EuroSCORE	7	4	0.02*

* Statistically significant.

In 11 patients (19.6%), endoleaks were detected during the procedure but no correlation was found between neck length and endoleak development (*R* = 0.01, *p* = 0.83). In patients with an observed endoleak during the procedure, even if treated, there was a significant correlation with mortality rate (OR = 6.6) (95% CI: 1.03–42.23).

## Discussion

Pre-operative diagnostic studies of EVAR patients are important. Implantation of the graft in the correct manner is directly related to the anatomy of the patient. It is known that patients with aneurysms whose neck length is longer than 1.5 cm, without a surrounding thrombus, and with limited angulation, are ideal cases for implementation of EVAR. However, with recent increased surgical experience and the improvement in graft technology, difficult cases can now also be treated with EVAR.^1^,^5^-^7^ It is therefore possible that, in our study, the pre-operatively measured aneurysm diameter, neck length and neck angle were not found to have an effect on mortality rate. However, the mortality rate was observed to be higher in patients with detected endoleaks, even though they were treated (*p* = 0.001).

We know that as the aneurysm diameter increases, the risk of rupture also increases. In their study, Brown *et al.*^2^ showed that the annual rate of rupture in aneurysms was 2.2%. The female gender, COPD, smoking and hypertension were indicated as risk factors for rupture.^2^ In the current study, urgent EVAR was performed due to aneurysm rupture in four of 56 patients and the success rate of treatment in these patients was 100%. All of the ruptured aneurysms were in group II and the correlation between the increase in aneurysm diameter and rupture was significant (*p* = 0.001).

In recent studies, it has been found that in all patients, not only in those with a high surgical risk, EVAR should be the firstline treatment method in the presence of suitable anatomical conditions.^1^-^7^ Brewster *et al.* found that the most important fatal complication in endovascular treatment was aneurysm rupture, which was seen at a rate of 1% following repair. The mortality rate was found to be the same in the surgical group one year after the procedure.^5^,^6^

In a large, randomised study in the United Kingdom, it was found that the early mortality rate was lower with EVAR; however, with long-term follow up, the mortality rate was found to be similar to that with surgical repair. Additionally, in the EVAR group, further additional interventions were required and more graft complications were observed.^9^

In their five-year EVAR follow up, Zarins *et al.* stated that small aneurysms (5 cm) responded best to treatment, and they reported the survival rate at 99%. With large aneurysms, rupture, deaths and additional surgical requirements were reported to be higher.^10^

In their study, Peppelenbosch *et al.* found that aneurysms with large diameters caused increased early and long-term mortality rates and risk of rupture when compared to aneurysms with intermediate and small diameters. The mortality rate increased, particularly after the fourth year.^11^ In our study, no correlation was found between diameter of aneurysm and mortality rate in the early to intermediate period.

In the study by Schanzer *et al.*, endoleak, advanced age, increased aneurysm neck angle (> 60°), and diameter of the main iliac artery larger than 20 mm were found to be independent risk factors for increase in aneurysm diameter following the procedure.^12^ However, there are no data related to the treated endoleaks at the conclusion of these studies.

Tsilimparis *et al.* showed that long-term survival of the AAA patients whose aortic diameter was less than 60 mm was superior to the ones whose aortic diameter was more than 60 mm.^13^ In our study, the diameter of the aneurysm had no effect on long-term survival. The factors affecting long-term survival were identified as high EuroSCORE values and endoleak formation during the procedure, even if it had been repaired.

In a single-centre retrospective review, Wisniowski *et al.* described age, American Society of Anesthesiologists (ASA) score, and chronic obstructive pulmonary disease as predictive factors for mortality at three years; and age, ASA score, renal failure and serum creatinine level as predictive factors for mortality after five years of follow up.^14^ They did not include aneurysm size as a predictive factor in their study. Similarly, Wang *et al.* found no significant difference in outcome of EVAR with small versus large AAA.^15^

There are some limitations to this research study. The first is related to the selection of stent types. We carried out this study with heterogeneous stent types in order to get initial preliminary results, avoiding bias. These results could be improved with homogenous stent types in further studies. The second limitation is related to follow-up period. Long-term data have not yet been obtained.

## Conclusion

As a result of our study we observed that pre-operative anatomical characteristics of the aneurysms did not increase mortality rate at a mean period of 50 months of follow up after treatment with EVAR. However, the complications that developed during and after the procedure increased the mortality rate. Moreover, we believe that early intervention would be more helpful, as confirmed rupture is more frequently seen in large aneurysms.
